# Playing cancer at its own game: activating mitogenic signaling as a paradoxical intervention

**DOI:** 10.1002/1878-0261.12979

**Published:** 2021-05-26

**Authors:** Matheus Henrique Dias, René Bernards

**Affiliations:** ^1^ Division of Molecular Carcinogenesis Oncode Institute The Netherlands Cancer Institute Amsterdam The Netherlands

**Keywords:** cancer therapy, collateral sensitivity, mitogenic signaling, stress response

## Abstract

In psychotherapy, paradoxical interventions are characterized by a deliberate reinforcement of the pathological behavior to improve the clinical condition. Such a counter‐intuitive approach can be considered when more conventional interventions fail. The development of targeted cancer therapies has enabled the selective inhibition of activated oncogenic signaling pathways. However, in advanced cancers, such therapies, on average, deliver modest benefits due to the development of resistance. Here, we review the perspective of a ‘paradoxical intervention’ in cancer therapy: rather than attempting to inhibit oncogenic signaling, the proposed therapy would further activate mitogenic signaling to disrupt the labile homeostasis of cancer cells and overload stress response pathways. Such overactivation can potentially be combined with stress‐targeted drugs to kill overstressed cancer cells. Although counter‐intuitive, such an approach exploits intrinsic and ubiquitous differences between normal and cancer cells. We discuss the background underlying this unconventional approach and how such intervention might address some current challenges in cancer therapy.

## Graphical abstract

Cancer cells are defective in homeostatic feedbacks. Therefore, pharmacological mitogenic stimulation disrupts the homeostasis of cancer cells and further increases their overall stress levels. Such toxic overactivation should increase the sensitivity of these cells to stress‐targeted drugs, providing opportunities for efficient drug combinations. Normal cells should more efficiently buffer such overactivation due to a more effective negative feedback network.

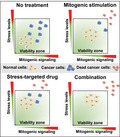

**Abbreviations**
ALL: acute lymphoblastic leukemia
DDR: DNA damage response
ESFT: ewing sarcoma family tumor
ER: endoplasmic reticulum
MAPK: mitogen‐activated protein kinase
PKC: protein kinase C
ROS: reactive oxygen species

## 1 Introduction

Genetic and epigenetic alterations leading to oncogenic signaling underlie the hallmarks of cancer and are also responsible for drug resistance [1, 2]. Most of the current targeted therapies are focused on blocking such aberrant mitogenic signaling pathways in cancer cells. However, the natural selection occurring throughout tumorigenesis shapes cancer cells toward the most efficient, not necessarily the highest, level of mitogenic signaling activity. This notion is becoming increasingly apparent, with accumulating evidence showing that overactivation of these same pathways might also disrupt cancer cell homeostasis. For instance, *EGFR* and *RAS* mutations are frequent but not concomitant in lung cancer. It has been recently shown that synthetic lethality rather than redundancy underlies this mutual exclusivity [3, 4]. Moreover, in a *Kras* (G12V)/*Braf* (D631A)‐driven lung cancer model, the ablation of the wild‐type *Braf* allele further increased mitogen‐activated protein kinase (MAPK) signaling, leading to oncogenic toxicity and preventing the development of adenocarcinoma [5]. Another case in point is that an unbiased loss‐of‐function genetic screen looking for chromatin modulators that induce senescence in melanoma cells revealed SMARCB1 as the top hit [6]. The knockout of this tumor suppressor gene in *BRAF* mutant melanoma cells triggered senescence and apoptosis through overactivation of the EGFR‐MAPK pathway [6]. Pharmacological examples of this oncogenic overload were also found in melanomas. A subgroup of *BRAF^V600E^*‐driven melanomas acquires resistance to BRAF inhibitors by overexpressing this protein. The inhibitor withdrawal results in tumor regression due to an overactivated MAPK [7]. In line with this, we previously showed that EGFR expression in melanoma models triggers oncogene‐induced senescence in the absence of drugs but becomes beneficial in the presence of BRAF or MEK inhibitors [8]. We later showed that BRAF inhibitor‐resistant melanoma cells have increased reactive oxygen species (ROS) levels due to overactivation in MAPK signaling. Subsequent addition of the histone deacetylase inhibitor vorinostat further increases the ROS levels, selectively killing these drug‐resistant tumor cells [9].

Collectively, these data highlight that too much oncogenic activity can be as lethal to cancer cells as the inhibition of oncogenic pathways. Based on this notion, we propose that a deliberate overactivation of mitogenic signaling pathways can be used to disrupt the fragile homeostasis of cancer cells. Such ‘paradoxical’ therapeutic intervention exploits the most basic characteristic of cancer cells, pathological oncogenic signaling, to push these cells over the viability edge.

Another ubiquitous feature of cancer cells is the increased mobilization of stress response pathways, which can be seen as an inherent cost for the oncogenic activity. Multiple cellular stress response pathways may have to be mobilized by the cancer cells to support the tumorigenic state. Together, they can be recognized collectively as the stress hallmarks of cancer [10]. The causes and consequences of these adaptations are interconnected but vary among different cancer cells. Nonetheless, the aneuploidy so characteristic to cancer cells is causally linked to proteotoxic, replication, mitotic, metabolic, and oxidative stresses, and the engagement of the respective stress response pathways [11]. Importantly, these adaptations are not merely associated with the malignant transformation but instead are intrinsically linked to it. Such increased mobilization and dependence on stress response pathways results from the upregulation of survival and mitogenic signaling in cancer cells. A dynamic and fine‐tuned balance between the hyperactivated signaling pathways and the stress response pathways induced by them supports cancer cell viability and tumor progression [10]. Indeed, the rationale for using drugs targeting stress pathways (referred to below as ‘stress‐targeted drugs’) has recently gained traction, showing encouraging results in disrupting the homeostasis of cancer cells [12, 13, 14, 15, 16].

**Fig. 1:**
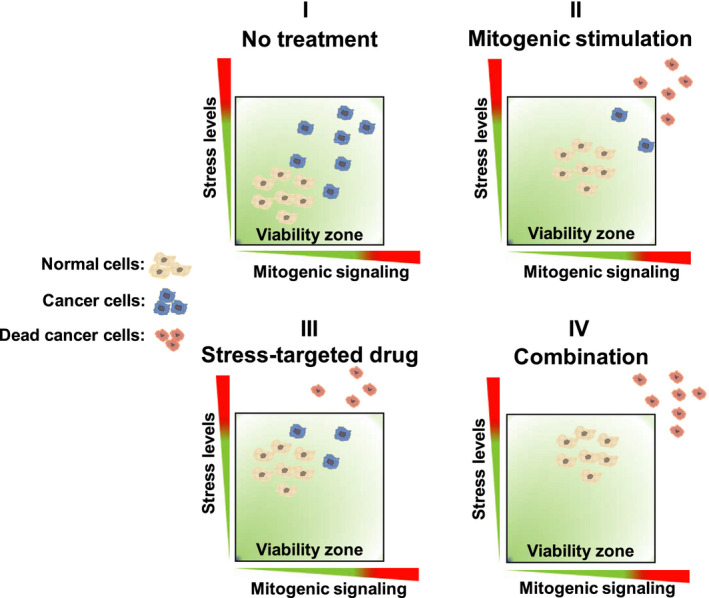
Schematic model of a paradoxical intervention for cancer treatment. (I) In untreated conditions, cancer cells show higher basal levels of mitogenic signaling and stress, inherent to the malignant phenotype, with a higher degree of heterogeneity in these levels. (II) Mitogenic stimulation takes advantage of the defective homeostatic feedbacks of cancer cells to promote overactivation of mitogenic signaling, driving part of the cancer cell population out of the viability zone and also increasing the overall stress levels. Normal cells, on the other hand, can buffer such activation due to an intact and effective negative feedback network. (III) Stress‐targeted drugs increase the overall stress levels and promote the killing of cancer cells based on their higher basal levels of stress; however, the heterogeneity of the cancer cell population allows some cancer cells to remain viable in the presence of the increased stress. (IV) The combination of mitogenic stimulation and targeting of stress response pathways should drive cancer cells to an overactivated, overstressed phenotype, out of the viability zone, promoting selective killing. Normal cells retain sufficient homeostatic feedback mechanisms to survive these perturbations.

The scenario outlined above suggests that further activation of mitogenic pathways should increase the dependency of cancer cells on stress response pathways. Therefore, we advocate that pharmacological overactivation of the same pathways driving tumorigenesis can disrupt the homeostasis of cancer cells and that such drugs may show synergy in combination with stress‐targeted drugs. A schematic model illustrating this rationale is described in Fig. 1. We address below how this paradoxical approach may have implications for different aspects of cancer treatment.

## 2 Selectivity: targeting the fundamental difference between normal cells and cancer

Highly selective targeting of cancer cells is mostly an elusive enterprise because they survive and proliferate using the very same signaling and metabolic pathways that normal cells employ to maintain homeostasis. Moreover, cancer cells also show genetic instability, self‐sufficiency in growth signals, and a lack of commitment to systemic homeostasis. Together, these properties provide them with the plasticity required to rewire signaling and adapt to drug treatment. These ‘hallmarks of cancer’ [1] are promoted by the activity of the driver oncogenes and the partial loss of counteracting tumor‐suppressive mechanisms that are the basis of cancer. Therefore, pathological mitogenic signaling and disruption of homeostatic feedback mechanisms represent a cornerstone difference between cancer and normal cells, forming the basis for selective targeting. Recent data, discussed below, show that deliberate reinforcement of mitogenic signaling disrupts the labile homeostasis and increases the mobilization of stress response pathways in cancer cells.

As one example, we recently showed that FGF2 enhances replication and proteotoxic stresses in human Ewing sarcoma family tumor models and *KRas*‐driven murine adrenal cancer cells. Indeed, the combination of exogenous FGF2 and the proteasome inhibitor bortezomib or inhibition of the DNA damage response (DDR) with the ATR inhibitor VE‐821 synergically triggered cell death, which was dependent on Ras‐Mek‐Erk signaling [17]. It is noteworthy that FGF2 inhibits endoplasmic reticulum (ER) stress *in vitro* and *in vivo*, protecting mouse cardiomyocytes from ischemia/reperfusion injury, also through Mek‐Erk activation [18]. These findings highlight opposite outcomes resulting from exogenous mitogenic signaling activation in normal versus cancer cells. In line with this, the EGF‐EGFR signaling axis is a model of prosurvival and proliferative signaling for normal cells, playing a key role in development, homeostasis, and wound healing [19]. In contrast, exogenous administration of EGF induced oxidative stress and PTEN upregulation in non–small‐lung cancer models, resulting in terminal autophagy and apoptosis *in vitro* and *in vivo* [20].

Hyperactivation of mitogenic pathways in tumor cells for therapeutic purposes is mostly uncharted territory. Therefore, the number of molecules other than growth factors that have been used in this context is quite limited. In this respect, protein kinase C (PKC) agonists have a substantial record of cytostatic and cytotoxic effects in multiple cancer models, often linked mechanistically to overactivation of the MAPK pathways [21, 22, 23] However, sustained activation of PKC isozymes leads to the downregulation of their protein levels [24], imposing a potential handicap for the therapeutic use of these molecules. Recently, Harold Varmus' laboratory showed that the small‐molecule DUSP1/6‐inhibitor BCI leads to toxic high levels of MAPK activity and triggers cell death in a panel of *Egfr*‐ and *Kras*‐driven lung adenocarcinoma cells [25]. It was later shown that the same inhibitor sensitizes ovarian cancer cells to carboplatin and paclitaxel [26]. These findings suggest that, although aberrant MAPK‐ERK1/2 activity is frequent in cancer cells, pharmacological DUSP inhibition and the resultant hyperactivation of this pathway can, in fact, be tumor suppressive. Indeed, Markus Müschen's laboratory has previously shown that DUSP6 activity is essential to tame ERK1/2 activity in pre‐B acute lymphoblastic leukemia (ALL) cells [27]. DUSP6 inhibition increases ROS levels and DDR activation, which triggers cell death in patient‐derived ALL cells [27]. More recently, the authors proposed the use of such therapeutic activation of ERK1/2 in STAT5‐driven B‐ALL based on the increased lifespan observed in patient‐derived xenografted mice treated with the DUSP1/6 inhibitor BCI‐215 to activate ERK1/2 [28]. It appears likely that combination with drugs targeting oxidative stress or DDR would improve the overall efficacy of the pharmacological ERK1/2 activation in these models.

**Fig. 2:**
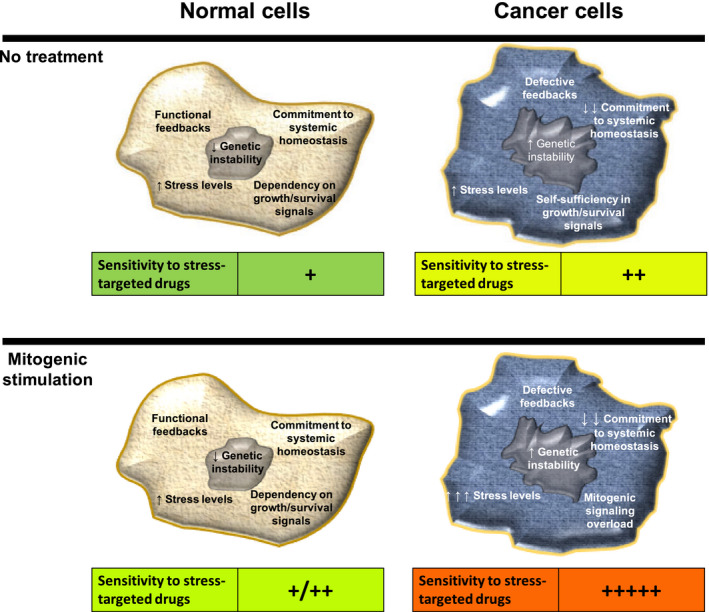
Conceptual rationale for the selective sensitization of cancer cells to stress‐targeted drugs through mitogenic signaling activation. (Top) Lack of commitment to systemic homeostasis, increased genetic instability, self‐sufficiency in growth and survival signals, and defective feedback signaling are recognized hallmarks of cancer cells and a cornerstone difference between cancer and normal cells. To support this tumorigenic state, cancer cells also show higher basal levels of stress and are therefore usually more sensitive to stress‐targeted drugs than are normal cells. (Bottom) Deliberate mitogenic stimulation would take advantage of the already increased mitogenic signaling of cancer cells and their defective feedbacks to promote mitogenic overload, while increasing the levels of stress and sensitivity to stress‐targeted drugs. On the other hand, normal cells display layers of feedback mechanisms to more efficiently tame such mitogenic activation, and lower basal levels of stress, being therefore potentially less sensitive to the combination of mitogenic activation and stress‐targeted drugs.

The obvious initial concern for using mitogenic signaling activation in combination therapies is that normal cells could become malignant themselves. However, normal tissues display layers of control to restrain unscheduled proliferation, such as a myriad of negative feedbacks in signaling pathways, terminal differentiation, and subordination to cell‐extrinsic tumor‐suppressive signals. A number of these controls are lost in cancer cells to allow the malignant phenotype. Supporting this notion, transgenic mouse models overexpressing growth factors [29, 30, 31, 32], or displaying tonic ERK1/2 activation due to DUSP6 knockout [33] do not show increased tumorigenesis. Therefore, reinforcement of mitogenic signaling exploits a fundamental defect of cancer cells (i.e., increased oncogenic signaling) and may be combined with stress‐targeted drugs. The specific toxicities resulting from the overactivation of mitogenic pathways certainly vary among different cancer cells and contexts. Nevertheless, further increase of genotoxic, oxidative, proteotoxic, mitotic, and metabolic stresses likely result from such intervention in cancer cells, making them more sensitive to drugs targeting these stress response pathways. Figure 2 outlines the rationale for the selective targeting of cancer cells through this proposed approach.

## 3 Drug resistance: exploiting ‘collateral sensitivities’ and promoting tumor‐suppressive acquired resistance

While chemo‐ and radiotherapy are based on killing rapidly dividing cells, the newer targeted therapies aim to selectively inhibit the very pathways that drive tumorigenesis [34]. Unfortunately, more often than not, targeted therapies fail to deliver long‐lasting therapeutic responses, as multiple mechanisms have been identified that can lead to resistance to these drugs [35, 36]. This is perhaps not surprising given the abundance of feedback loops and cross‐talks that operate between cellular signaling pathways, providing ample opportunity for cells to rewire their signaling under selective pressure. Massive efforts have been made to uncover powerful drug combinations, or ‘synthetic lethalities’, to tackle signaling rewiring and constrain drug resistance [37, 38]. Despite some progress in this endeavor, good long‐term prognoses are still not frequent for advanced cancer patients. The rationale behind targeted therapies is that cancer cells are dependent on specific driver signaling pathways whose inhibition would result in cell cycle arrest or death [39]. However, genetic and epigenetic plasticity at the cell level and the frequent heterogeneity at the tumor level make the bypass of these inhibitions often only a matter of time. The survival and proliferation of normal cells also rely on many of the same pathways targeted by antimitogenic drugs, but their commitment to systemic homeostasis gives them less ‘freedom’ to bypass antimitogenic therapies [40].

Exploiting the acquired vulnerabilities/fitness costs inherent to the drug‐resistant phenotype holds promise for the treatment of drug‐resistant cancers. This concept of ‘collateral sensitivity’ was recognized decades ago [41], and the recent literature has demonstrated its utility to outsmart drug resistance [42]. The attractiveness of the collateral sensitivity approach is the use of the very plasticity of cancer cells to steer them toward a predictable and targetable, highly vulnerable, phenotype. In this sense, the use of mitogenic activation to make cancer cells more targetable by stress‐related drugs may be one embodiment of the collateral sensitivity approach. The exploitation of such a frequent feature of cancer cells (i.e., overactivation of mitogenic pathways) to induce a collateral sensitivity may provide a more homogeneous sensitization of the cancer cells and thus lead to more robust responses when the collateral sensitivity is targeted. The coincident targeting of an equally ubiquitous feature (i.e., increased dependency on stress response pathways) can represent a powerful combination strategy. The specific activation signal and stress‐targeted drug in such a combination will most likely vary for different cancer phenotypes. Nonetheless, in contrast to antimitogenic therapies, this approach benefits from the fact that different mutations driving human cancers converge on a much smaller set of signaling pathways. This notion implies that different upstream or downstream activators can promote the same mitogenic signaling pathway, resulting in a similar collateral sensitivity.

One of the most attractive features of the paradoxical cancer therapy proposed above comes to light when considering how cells might develop resistance to this type of therapy. Resistance to antimitogenic targeted therapies often involves reactivation of the inhibited pathway or activation of parallel oncogenic signaling [43]. Hence, it is reasonable to assume that resistance to overactivation of mitogenic signaling pathways will develop in the opposite direction. That would include the upregulation of tumor‐suppressive mechanisms and downregulation or even loss of oncogenic signaling. Striking data in line with these assumptions come from the virtual absence of *EGFR* amplification in glioblastoma cell models *in vitro*, whereas it is found in about half of the diagnosed glioblastoma tumors [44]. Schulte and coworkers showed that the presence of EGF in the culture media purges *EGFR* gene amplification from patient‐derived glioblastoma cells, largely decreasing their tumorigenicity in xenografts [45]. That might be a stark example of a tumor‐suppressive effect (i.e., loss of *EGFR* amplification) triggered by mitogenic signaling stimulation (i.e., EGF ligand), but it is not without precedent, given that similar findings were previously described for lung [46] and breast [47] cancer models.

The overall scenario discussed above raises the prospect that ‘tumor‐suppressive drug resistance’ might turn cancer into a chronic disease, a long‐awaited therapeutic goal. This assumption may seem unrealistic at first glance; nonetheless, deep‐sequencing investigations have recently revealed that clones carrying known driver oncogenic mutations are present in clinically normal skin, esophagus, and endometrium tissues, among others [48, 49, 50]. Moreover, it has long been shown that highly malignant teratocarcinoma cells injected into blastocysts resulted in mosaic mice, with a substantial contribution of the tumor‐derived cells to the normal functions of many tissues [51]. Similar reprogramming was also described for melanoma cells in different embryo models [52]. It remains to be addressed whether the relative higher abundance of mitogenic signals in embryos compared with aged or adult tissues is required for this tumor‐suppressive reprogramming.

## 4 Dormancy and relapse: restraining nonproliferative drug tolerance and resistance

The therapeutic window for cancer treatment often relies on a common trait of malignant cells: unscheduled and fast proliferation resulting from the effect of driver oncogenes on mitogenic pathways. Unfortunately, tumor heterogeneity and plasticity often make it possible for a fraction of the tumor cell population to acquire a nonproliferative drug‐tolerant phenotype under treatment. This shift is now recognized as a common mechanism underlying acquired resistance and tumor relapse [53, 54, 55]. The transition to these nonproliferative phenotypes involves rewiring signaling pathways to evade the oncogenic signaling and its proliferative consequences. Moreover, adjustments in the fine‐tuned cross‐talk between mitogenic signaling and stress response pathways support the viability of nonproliferative drug‐resistant cancer cell phenotypes (i.e., quiescent, dormant, persister, and cancer stem cells) [56, 57, 58]. Therefore, the combination of mitogenic signaling activation with stress‐targeted drugs may hinder therapy escape by restraining the exit of cancer cells from the pathologically proliferative phenotype.

In agreement with this rationale, mitogenic stimulation of quiescent drug‐resistant leukemic cells has been shown to drive them back to the proliferative phenotype and sensitize to chemo‐ and targeted therapies [59]. Even in the context of cancer stem cells, a similar approach stimulated leukemia stem cells into the cell cycle, sensitizing them to the standard‐of‐care chemotherapy in a xenotransplantation mouse model [60]. Cancer stem cells represent a formidable hurdle for cancer therapy. They share many features with normal stem cells, such as self‐renewing capability, pluripotency, and cell surface markers [61]. More importantly, they also have in common slow proliferation rates and overall metabolism, high expression of drug efflux pumps, and a consequent multidrug‐resistant phenotype [61, 62]. Not surprisingly, cancer stem cells are very elusive targets for current cancer therapies, being a reservoir for tumor relapse and metastasis after therapy withdrawal or acquired resistance [63, 64].

There is a lack of consensus about the origin, prevalence, and markers of cancer stem cells and other nonproliferative drug‐resistant phenotypes. Even so, in practical terms, they may represent a metastable state in which selective targeting of cancer cells becomes unfeasible. As such, the use of mitogenic stimulation as part of the therapy holds the promise to push cancer stem cells toward a proliferative phenotype. By doing this, we make them more different from normal cells, more prone to drug toxicity, and prevent them from hiding from therapeutic attack.

## 5 Side effects: targeting cancer cells while supporting tissue homeostasis and a tumor‐suppressive microenvironment

It is now clear that healthy normal cells and tissue homeostasis play a pivotal role in preventing tumor onset and progression, even when clones carrying known driver oncogenic mutations are frequent [65, 66]. This notion underlies the known correlation between tumorigenesis and aging, inflammation, chemical injuries, or other conditions leading to decreased tissue homeostasis. The fact that growth factors and mitogenic signaling drive recovery from injury is documented extensively [67, 68]; additionally, the contribution of these signaling pathways for tissue homeostasis becomes evident by the many on‐target toxicities associated with tyrosine kinase inhibitors [69]. This overall scenario suggests that therapeutic mitogenic signaling activation may not only sensitize cancer cells to stress‐targeted drugs but also favor a tumor‐suppressive microenvironment by improving tissue homeostasis.

In agreement with the assumptions above, FGF signaling promoted self‐renewing proliferation and inhibited senescence in normal stem cell pools from various tissues [70]. From the regenerative perspective, regular FGF2 injections promoted neurogenesis and increased the lifespan in neurodegenerative Huntington's disease mouse models [71]. Similarly, daily EGF administration following total body irradiation dramatically improved the recovery of the hematopoietic stem cell pools and overall survival in mice [72]. In this context of therapy models, systemic administration of human recombinant EGF in mice reduced the severity of the oral mucositis associated with chemotherapy and radiotherapy for head and neck cancers [73]. Debilitating oral mucositis is indeed a frequent side effect in patients receiving myelotoxic therapy [74]. KGF (FGF7) has been used in the clinic, decreasing the severity and shortening the recovery of the lesions [75]. Systemic homeostatic effects were described for FGF21, which protects different organs against tissue damage resulting from metabolic disorders and metaflammation, promoting tissue homeostasis [76]. It is noteworthy that the homeostatic effects of FGF21 in nonmalignant tissues also involve the activation of the very same FGFR‐MAPK axis frequently overridden in cancer cells [77].

Collectively, the findings above highlight that normal cells rely on the abundance of mitogenic signaling to maintain tissue homeostasis and recover from injury, and imply that pharmacological mitogenic signaling activation might be clinically safe. This dependency on mitogenic signaling in normal cells contrasts with the self‐sufficiency in survival and proliferation signals characteristic to cancer cells. Different conditions leading to decreased mitogenic signaling levels in normal cells should, therefore, impair tissue homeostasis and cell‐extrinsic tumor‐suppressive mechanisms. In line with this, the progressive loss of tissue homeostasis with aging is associated with decreased levels of mitogenic signals [78, 79, 80, 81]. These findings indicate that mitogenic signaling is inversely correlated with the major risk factor for cancer incidence [82]. These observations may puzzle our notion of the role of growth factors and mitogenic signaling for cancer onset and progression. However, this overall decline in mitogenic signals, detrimental to normal cells, likely drives the competition in favor of precancer clones carrying oncogenic mutations. Hence, if cancer is ‘a wound that does not heal’ [83], combining ‘healing signaling’ and stress‐targeted therapies might revert this pernicious dynamic, improving several aspects of cancer treatment.

## 6 Future perspectives

While counter‐intuitive at first glance, there appears to be a therapeutic window to selectively kill cancer cells based on the further activation of an already activated oncogenic signaling pathway. This approach opens a number of ‘out of the box’ treatment options in cases in which conventional therapies have failed. Here, we have focused on mitogenic signaling pathways, because these pathways are often the target of oncogenic mutations. However, the concept of ‘making a bad situation worse’ can also be applied to other treatment modalities. For instance, along the same lines discussed above for mitogenic signaling, it has been proposed that the level of genetic instability, a common feature of malignant transformation, is optimal, not maximal, in cancer cells [84, 85]. Deliberate overactivation of DDR signaling has been proposed to decrease the ability of cancer cells to deal with DNA damage [86]. Moreover, because cancer is a disease that results from mutations, it would seem illogical to conceive of a cancer therapy that is based on increasing the mutation burden of a cancer cell. Nevertheless, there is emerging evidence that responses to checkpoint immunotherapies can be enhanced by increasing the mutation load of the cancer. This approach is based on the notion that there is a striking correlation between the mutation burden of a given cancer and the response to these checkpoint therapies [87]. Thus, microsatellite instable colon cancers, which have a higher mutation burden due to a defect in DNA mismatch repair, show superior responses to immunotherapies as compared to microsatellite stable colon tumors [88, 89]. One paradoxical intervention may therefore be to increase the mutation burden in a microsatellite stable colon cancer by disabling the mismatch repair machinery to increase the neo‐antigen load of the cancer. Indeed, Germano and coworkers showed that genetic inactivation of the *MutL* homolog *MLH1* leads to increased mutation burden, immune recognition, and response to checkpoint therapies [90]. Along these same lines, Bartok *et al*. [91] recently demonstrated that amino acid starvation can result in ‘ribosomal frameshifting’, leading to generation of neoantigens that can be seen by immune cells.

Finally, it has been suggested that deliberate interference with mRNA splicing can serve as a source of cancer neoantigens [92]. This is potentially interesting, given that drugs exist that interfere with mRNA splicing [93]. Thus, in the context of the immune response, the paradoxical intervention could be the reinforcement of the ‘non‐self’ character of tumor cells while favoring an active cytotoxic phenotype in T cells. This scenario would increase the overall immunogenicity of cancer cells, potentially acting to enhance the effects of current immunotherapies.

We have argued above that such paradoxical interventions may have only modest effects on normal cells. However, it should be borne in mind that most of the experiments to test such interventions on normal cells have been carried out in mice. Two crucial differences between mice and humans are relevant in this context: mice weigh 2500‐fold less than an average human (and have correspondingly fewer cells) and their lifespan is 40x shorter than that of humans. Thus, it may be dangerous to translate the effects of mitogenic insults on normal cells observed in mice to the human situation. There may be late effects in humans of this type of paradoxical therapy that were never seen in simple animal models. Notwithstanding this limitation, we argue here that there are compelling data from a number of independent models to seriously consider this as a novel approach to cancer therapy.

## Conflict of interest

The authors declare no conflict of interest.
